# Neuropeptide S pathway in PTSD and neuropsychiatric disorders: A review

**DOI:** 10.17305/bb.2025.12861

**Published:** 2025-08-04

**Authors:** Zhi-cheng Zhu, Xue-jing Han, Zhen He, Meng-yang Liu, Ning Wu, Xiang-min Tong, Fei Li

**Affiliations:** 1 Beijing Institute of Pharmacology and Toxicology, Beijing, China; 2 Department of Pharmacology, Hangzhou Normal University, Hangzhou, Zhejiang, China; 3 Department of Pharmacy, Shenyang Pharmaceutical University, Shenyang, Liaoning, China; 4 Department of Hematology, Zhejiang Provincial People’s Hospital and People’s Hospital of Hangzhou Medical College, Hangzhou, China

**Keywords:** Post-traumatic stress disorder, related neuropsychiatric disorders, neuropeptide S, neuropeptide S receptor, single nucleotide polymorphism

## Abstract

Post-traumatic stress disorder (PTSD) is a multidimensional illness that seldom occurs alone: roughly 80% of patients also meet criteria for anxiety, depression, chronic pain, substance-use, eating or cognitive disorders. Converging genetic, neurochemical and behavioral findings implicate the neuropeptide S (NPS) system—acting through its G-protein-coupled NPS receptor (NPSR)—as a common regulator of these diverse phenotypes. This narrative review surveys studies published 2000–2024 in PubMed, Embase and Web of Science that examine NPS/NPSR involvement in core PTSD features and typical comorbidities. The functional rs324981 A/T polymorphism, which boosts NPSR surface expression and signaling, consistently associates with greater PTSD risk and symptom severity. In rodent models, exogenous NPS reduces anxiety- and fear-like behaviors, speeds fear-memory extinction, stabilizes the hypothalamic–pituitary–adrenal axis, enhances dopaminergic tone and elevates hippocampal brain-derived neurotrophic factor (BDNF)—changes concordant with symptom relief. Additional work shows that NPS lessens pain affect, dampens alcohol and opioid intake, eases withdrawal-induced anxiety and lowers food consumption, hinting at a multimodal therapeutic profile. These effects converge on limbic and mid-brain circuits (amygdala, ventral tegmental area, locus coeruleus, paraventricular nucleus) and engage oxytocinergic, adenosinergic and endocannabinoid pathways. Translation remains limited by NPS’s rapid degradation, poor blood–brain-barrier penetration and scarcity of brain-penetrant NPSR ligands, but advances in intranasal delivery, lipid-acylated analogs, biased NPSR agonists and “humanized” NPSR-variant models offer promising solutions. Collectively, current pre-clinical and genetic evidence positions the NPS–NPSR axis as a versatile therapeutic target for both core PTSD symptoms and their disabling comorbidities, warranting rigorous translational studies to refine mechanism, optimize drug-like properties and test clinical efficacy.

## Introduction

Post-traumatic stress disorder (PTSD), also referred to as delayed psychogenic reactions, is a psychological condition resulting from exposure to significant trauma or catastrophic events. This disorder is characterized by delayed onset and can lead to enduring mental health issues. PTSD has emerged as a prevalent mental health concern, with incidence rates of approximately 6%–9% in the general population [1, 2]. However, among individuals who have experienced severe trauma—such as combat veterans, refugees, victims of assault, and those facing pandemic-related stressors—the prevalence may rise to approximately 25%. During the COVID-19 pandemic, studies indicated that between 15.8% and 35.6% of the U.S. population reported symptoms of PTSD [3], while 10.7% to 23.5% of frontline healthcare workers developed the disorder [4, 5]. Classic symptoms of PTSD primarily include the re-experiencing trauma, avoidance behaviors, negative emotions and thoughts, and hyperarousal. It is important to recognize that individuals exposed to complex traumatic events are not only at risk for developing PTSD but often exhibit comorbid conditions such as depression [6], anxiety [7], insomnia [8], cognitive impairment [9], chronic pain [10], substance abuse [11], and eating disorders [12]. Epidemiological studies indicate that approximately 80% of individuals with PTSD meet the criteria for at least one additional psychiatric diagnosis, including depression and substance use disorders [13]. Consequently, the treatment of PTSD can be particularly challenging, especially in the context of concurrent comorbidities. In recent years, significant attention has focused on the regulatory role of the endogenous substance neuropeptide S (NPS) within the central nervous system [14]. NPS is a polypeptide composed of 20 amino acids, exhibiting a highly conserved primary structure across vertebrate species. Its name derives from the presence of serine as the amino-terminal residue in NPS across various organisms [15]. The NPS receptor (NPSR), formerly designated as GPR154, functions as a double-coupled receptor linked to Gs and Gq proteins. When NPS binds to NPSR, it triggers the intracellular accumulation of cyclic adenosine monophosphate (cAMP) and facilitates the release of intracellular Ca^2+^, thereby activating the downstream mitogen-activated protein kinase signaling pathway [16].

The NPSR gene contains an A/T single nucleotide polymorphism (SNP rs324981) located at the 7P14 locus on chromosome 7, which results in an amino acid substitution (Asn107Ile) [17]. This mutation enhances the cell-surface expression of NPSR, rendering NPSR Ile107 approximately ten times more potent than NPSR Asn107, without affecting binding affinity [18, 19]. Haxhibeqiri et al. [20] demonstrated that the risk of PTSD is significantly elevated in homozygous T allele carriers compared to homozygous A allele carriers within the Balkan war population. Furthermore, NPSR rs324981 influences stress levels [21], the incidence of anxiety disorders [22, 23], alcohol use disorders (AUDs) [24], and cognitive impairments [25], all of which are comorbid symptoms of PTSD. Moreover, accumulating research indicates that NPS has the potential to alleviate PTSD-like behaviors, exert anxiolytic effects [26], diminish fear-related responses [27], improve learning and memory deficits [28], and regulate substance use disorders [29–31] (see Table 1). This body of evidence suggests that the NPS–NPSR system may play a critical role not only in the regulation of PTSD but also in related neuropsychiatric disorders.

**Table 1:** Effects of NPS on the animal behaviors associated with PTSD and its related neuropsychiatric disorders

**Animal**	**Active doses and route of administration**	**Symptoms**	**Experiments**	**Effects**	**Ref**
SD rats	NPS (1.0 nmol/0.5 µL, intra-BLA)	PTSD-like behaviors	Predator scent stress, elevated plus maze, cut-off behavioral criteria model, acoustic startle response, freezing behavior	Alleviate the PTSD-like behaviors, including anxiety, freezing response, and hyperarousal	[36]
C57BL/6J	NPS (10 µM/0.5µL, intra-LA)	PTSD-like behaviors	Immobilization stress, elevated plus maze, fear conditioning	Alleviate anxiety, reduce conditioned fear responses	[38]
Wistar rats	NPS (1 nmol, i.c.v.)	Aggressive and anxiety behaviors	Resident-intruder test, elevated plus maze	Alleviate anxiety, reduce aggressive behavior	[41]
Swiss mice, NPSR^(+/+)^ and NPSR^(−/−)^ with CD-1 strain	NPS (0.01–1 nmol, i.c.v.)	Aggressive behaviors	Resident/intruder test	Reduce aggressive behavior	[42]
Wistar rats	NPS (0.05 and 0.5 nmol/side, intra-VTA)	_	Locomotion	Increase locomotor activity	[59]
SD rats	NPS (1 nmol, i.c.v.)	Sleep deprivation and anxiety	Paradoxical sleep deprivation, open field test, light-dark box	Reduce paradoxical sleep deprivation induced anxiety-like behavior	[79]
NPSR^(+/+)^/NPSR^(−/−)^ mice with 129S6 genetic background	NPS (0.1 nmol, i.c.v.)	Anxiety	Locomotor	Increase locomotor activity in NPSR^(+/+)^ mice but not in NPSR^(−/−)^ mice	[90]
NPSR ^(+/+)^/NPSR^(−/−)^ with CD-1 genetic background	NPS (1 nmol, i.c.v.)	Anxiety	Elevated plus maze, open field test	Alleviate anxiety in NPSR^(+/+)^ mice but not in NPSR^(−/−)^ mice	[91]
SD rats	NPS (10 nmol, i.c.v.)	Pain	Hot plate, tail flick	Prolong the hot plate latency but not tail flick latency	[102]
Swiss mice	NPS (0.1 nmol, i.c.v.)	Pain	Formalin test	Reduced formalin-induced nociception	[103]
C57BL/6J	NPS (0.3 and 1 nmol, i.c.v.)	Pain	Hot-plate test	Prolong the hot plate latency	[104]
Alcohol-preferring and non-preferring rats	NPS (0.075, 0.3, and 1.2 nmol, i.c.v.)	Substances use disorder	Alcohol preferences, elevated plus-maze	Reduced alcohol consumption in the alcohol-preferring rat	[107]
Alcohol-preferring and nonpreferring rats	NPS (0.1, 0.5, 1.0, and 2.0 nmol, i.c.v.)	Substances use disorder	Alcohol self-administration	Reduced alcohol self-administration	[108]
Wistar rats	NPS (1.0, 2.0, 4.0 nmol, i.c.v.)	Substances use disorder	Alcohol self-administration	Increase ethanol seeking elicited by ethanol-associated cues	[109]
Kunming strain mice	NPS (1, 3, 6 and 10 nmol, i.c.v.)	Substances use disorder	Conditioned place preference	Reduce the morphine-induced CPP acquisition and expression	[111]
C57BL/6	NPS (1 nmol, i.c.v.)	Cognition deficit	Inhibitory avoidance, novel object recognition	Enhance memory retention, but not acquisition or recall	[120]
Kunming strain mice	NPS (1 nmol, i.c.v.)	Cognition deficit	Morris water maze	Facilitate spatial memory, mitigate spatial memory impairment	[121]
Adult male Wistar rats	NPS (1 nmol, i.c.v.)/Nasal NPS administration (40 nmol)	Cognition deficit	Elevated plus-maze, object discrimination, social discrimination	Reduce non-social anxiety, facilitate object discrimination but not social discrimination	[122]
C57BL/6	Nasal NPS administration (10 µL of 1 mM solution)	Cognition deficit	T-Maze	Facilitate reversal learning without affecting the acquisition	[123]
Kunming strain mice	NPS (0.1 nmol, i.c.v.)	Food intake in fasted mice	Food intake in fasted mice	Inhibit food intake in fasted mice	[134]

The doses of NPS presented in the table are statistically effective for alleviating the corresponding symptoms (*P* < 0.05). Abbreviations: i.c.v.: Intracerebroventricular infusion; intra-LA: Intra-lateral amygdala; intra-BLA: Intra-basolateral amygdala; intra-VTA: Intra-ventral tegmental area.

This review synthesizes evidence on the role of NPS–NPSR system in: (i) core PTSD symptoms, (ii) PTSD-associated neuropsychiatric syndromes, ultimately evaluating its potential as a multimodal therapeutic agent. We analyzed findings from studies published between 2000 and 2024, identified through systematic searches of PubMed, Embase, and Web of Science. The keywords employed included “PTSD,” “comorbidity,” “animal models,” “neuropeptide S,” “neuropeptide S receptor,” “single nucleotide polymorphism,” “anxiety,” “substance use disorder,” “pain,” “food intake,” “norepinephrine,” “dopamine,” and “hypothalamic–pituitary–adrenal axis” (HPA). We prioritized original research articles, reviews, and meta-analyses published in English, focusing on studies that investigate the role of NPS in modulating PTSD and related neuropsychiatric disorders. Studies that were irrelevant to the scope or had significant methodological limitations were excluded.

### Role of NPS–NPSR system in PTSD

Anxiety, conditioned fear responses, fear memory disorders, and severe stress reactions are the primary characteristics of PTSD [32, 33]. An expanding body of literature underscores the role of the NPS system in regulating stress, mood, cognitive function, and anxiety. Multiple studies have highlighted the amygdala’s crucial involvement in PTSD [34]. Neuroimaging studies of patients with PTSD have revealed increased activation in the amygdala [35]. Exposure to predator-scent stress (PSS) induces PTSD-like behaviors in rats. Microinjection of NPS into the basolateral amygdala (BLA) has been demonstrated to alleviate anxiety, reduce the freezing response, and restore the diminished expression of brain-derived neurotrophic factor (BDNF) and neuropeptide Y Y1 receptor (NPY-Y1R) in the hippocampus in this model [36]. Ten days post-experience of acute immobilization stress, mice exhibited a significant increase in PTSD-like behaviors, characterized by heightened anxiety and fear responsiveness [37]. This increase could be mitigated by injecting NPS into the lateral amygdala (LA), while administration of NPSR antagonists exacerbated the symptoms [38]. Jiang et al. [39] reported a significant reduction in NPSR mRNA levels in the hypothalamus of mice subjected to a single prolonged stress (SPS) model, while NPS mRNA levels were significantly elevated compared to the control group. Additionally, the repeated social defeat stress (R-SDS) model serves as an effective animal model for studying PTSD [40]. Within this framework, i.c.v. infusion of NPS (1 nmol) was shown to reduce anxiety and aggressive behaviors in both low and high anxiety rats [41]. Moreover, in the resident-intruder test—another model for PTSD-like behavior [37]—resident mice lacking NPSR exhibited longer attack durations on intruder mice than wild-type (WT) counterparts. Administration of NPS (0.01 nmol–1 nmol, i.c.v.) significantly decreased the number of attacks and the total time spent attacking by resident mice [42]. These findings suggest that the NPS system plays a pivotal role in modulating behaviors associated with PTSD.

### Possible mechanisms of NPS in PTSD

Norepinephrine (NE) plays a critical role in the body’s response to fear and anxiety. Symptoms of hyperawareness and irritability in patients with PTSD are associated with heightened NE activity [43]. Additionally, individuals with PTSD demonstrate increased cortisol and NE responses to stress [44, 45]. Clinically, the α1 receptor antagonist prazosin and the β receptor antagonist propranolol have shown potential in treating PTSD [43, 46]. Animal studies indicate that a single injection of isoproterenol, a nonselective β-adrenergic agonist, into the amygdala enhances the reconsolidation of fear memories, suggesting that elevated NE during fear memory processing may contribute to the persistence of traumatic memories [47]. NE is known to regulate the cAMP/PKA and CaMKII/PKC signaling pathways through the activation of β-adrenoceptors, which can result in PTSD-like memory impairments [47]. Giustino et al. [48] found that stress or activation of the locus coeruleus (LC)-NE pathway induces deficits in fear memory extinction. This effect is likely mediated by stress-induced increases in BLA activity, with intra-BLA administration of propranolol blocking the extinction deficits caused by LC-NE activation. Moreover, NE neurons in the LC have been found to express at least 19 neuropeptide transcripts [49]. NPS interacts with various neuromodulators, including NE [50]. Reports indicate that NPS inhibits NE release; specifically, Raiteri et al. [51] demonstrated that NPS selectively inhibits NE release through its action on noradrenergic nerve terminals within the frontal cortex. These findings suggest that NPS may reduce the activity of noradrenergic neurons by suppressing NE release, potentially aiding in the alleviation of fear memory reconsolidation and extinction. Dopamine (DA), the predominant catecholamine neurotransmitter in the brain, plays a crucial role in regulating various physiological processes, including reward, motivation, exercise, and emotion. Previous research indicates that dopaminergic dysfunction may directly contribute to impaired fear extinction, learning, and memory in patients with PTSD [52]. Animal studies have shown decreased DA levels in the medial prefrontal cortex (mPFC) and the BLA of rats following 18 days of exposure to traumatic stimuli in the SPS model [53]. Additionally, D2 receptors in the mPFC and BLA are implicated in fear extinction, as reduced receptor levels have been observed in rats during this process [54]. Notably, D1 receptor knockout mice do not exhibit fear memory in fear-conditioning experiments, suggesting the involvement of the dopaminergic system in fear learning and extinction [55]. Clinical studies have demonstrated that the DA receptor agonist kb220z significantly alleviates nightmare symptoms in PTSD patients [56]. Preclinical investigations have also revealed that DA receptor D2/D3 agonists, such as rotigotine and pramipexole, can diminish PTSD-like symptoms in experimental models [57]. NPS has been reported to enhance DA release, with i.c.v. administration of NPS leading to a dose-dependent increase in DA release in the rat mPFC [58]. Furthermore, microinjection of NPS into the ventral tegmental area (VTA) of rats has been shown to elevate locomotor activity and DA metabolites in the nucleus accumbens (NAc) [59]. Although the mechanisms of NPS in PTSD remain uninvestigated, these findings suggest that NPS may target the DA system to exert a potential anti-PTSD effect by increasing DA levels.

The HPA axis is a neuroendocrine system that regulates the body’s response to stress [60]. Key neuroendocrine hormones involved in the HPA axis include corticotropin-releasing hormone, adrenocorticotropic hormone, and cortisol [61]. Research indicates a strong association between HPA axis dysregulation and PTSD [62]. Numerous studies have documented this dysregulation in individuals with PTSD, characterized by increased sensitivity of the HPA feedback system, reduced cortisol levels, and diminished urinary cortisol excretion. These alterations contribute to symptoms such as fatigue and mood disturbances [63]. Animal research indicates that on the 18th day post-exposure to stress in the rat post-stress susceptibility (PSS) model, there is a significant reduction in basal corticosterone pulse amplitude and a dampened corticosterone response to stressors. This suggests that impairment in the corticosterone response may serve as a susceptibility or risk factor for the development of PTSD [64]. Furthermore, the administration of corticosterone to rats one hour after trauma stimulation diminishes fear memory retrieval and facilitates the extinction of fear memories in PTSD models [65, 66]. Elevated *in vivo* release of NPS in the amygdala has been observed following local depolarization and emotional stress [67]. Administration of NPS activates the HPA axis, resulting in the release of corticotropin-releasing hormone, adrenocorticotropic hormone, and corticosterone [68]. This cascade of hormonal events may play a critical role in the modulation of stress and anxiety by NPS [68, 69]. These findings suggest that targeting the modulation of the HPA axis through NPS may represent a promising approach for PTSD treatment. BDNF is the most prevalent neurotrophic factor in the body. Upon binding to its specific receptor, Tropomyosin-related kinase receptor B (TrkB), BDNF plays a crucial role in modulating synaptic plasticity, neuronal survival, and apoptosis [70]. Disruptions in the BDNF-TrkB signaling pathway may significantly impact PTSD [71]. In the hippocampus, impaired conditional-fear learning and extinction have been linked to the absence of BDNF-TrkB signaling in key regions, including the ventromedial prefrontal cortex (PFC), anterior cingulate cortex, and NAc [72]. Clinical studies have demonstrated a substantial reduction in BDNF levels in the blood of PTSD patients compared to healthy individuals, indicating its potential as a diagnostic biomarker for PTSD [73]. In the SPS rat model, a significant reduction in cerebrospinal fluid BDNF levels has been observed [74]. Furthermore, the administration of BDNF (1 mmol, 10 mmol, 30 mmol, i.c.v.) in this model has been demonstrated to dose-dependently increase the time spent in the central area relative to the total time in the open field test [74]. Additionally, research indicates that BDNF expression is decreased following PSS exposure but is enhanced after NPS microinjection into the BLA [36], suggesting that NPS may regulate neuronal plasticity and other functions through the modulation of BDNF.

### Role of NPS–NPSR system in the neuropsychiatric disorders related to PTSD

#### Role of NPS–NPSR system in anxiety disorder and implications for PTSD treatment

PTSD has a lifetime prevalence and shares neurobiological characteristics with anxiety disorders [7]. The five-year recurrence rate for PTSD among individuals with anxiety and depressive disorders is 9.2% [75], while the comorbidity rate of PTSD and social anxiety disorder (SAD) ranges from 14.8% to 46% [76]. Research indicates that plasma NPS levels are significantly elevated in individuals with generalized anxiety disorder (GAD) compared to healthy controls, suggesting a correlation between plasma NPS levels and anxiety symptoms in GAD patients. This finding implies that plasma NPS may serve as a potential biomarker for distinguishing GAD [69]. Various experimental paradigms have been utilized to investigate the effects of NPS on anxiety. Administration of NPS (1 nmol, i.c.v.) has demonstrated anxiolytic effects in numerous tests, including the open field test, elevated plus maze, marble-burying test, four-plate test, elevated zero maze, and stress-induced hyperthermia [15, 77]. Bilateral microinjection of NPS at a dose of 1 nmol, administered intracerebroventricularly (i.c.v.), into the amygdala of mice significantly mitigated anxiety-related behaviors in both the open field test and the elevated plus maze, suggesting an anxiolytic effect of NPS [78]. Conversely, the application of the NPSR antagonist SHA68 into the amygdala reduced the anxiolytic effects of NPS [15, 78]. Xie et al. [79] demonstrated that NPS administration (1 nmol, i.c.v.) alleviated anxiety-like behavior induced by paradoxical sleep deprivation, significantly enhancing the expression levels of NPSR mRNA and increasing the number of Fos-immunoreactive neurons in the BLA, central amygdala, and medial amygdala (MeA). Regarding the anxiolytic mechanisms of NPS, research has identified that its effects are linked to the activation of oxytocin (OXT) neurons within the paraventricular nucleus (PVN). Grund et al. [80, 81] found that NPS selectively activates a subset of OXT neurons in the PVN via NPSR. This activation promotes the local release of OXT and induces a transient increase in intracellular calcium concentration in OXT neuronal subgroups, leading to an anti-anxiety effect. Pharmacological blockade of local OXT receptors (OXTR), along with the silencing of OXT neurons through chemogenetic methods, inhibited the anxiolytic responses elicited by NPS. Furthermore, NPS influences synaptic events in key brain regions, such as the dorsal raphe nucleus (DRN) and the lateral dorsal tegmental (LDT) area, modulating neuronal excitability in these regions and contributing to the anxiolytic and arousal-promoting effects of NPS [82].

NPSR activity, as a target of NPS, is influenced by various environmental and physiological factors, including stress stimuli. Numerous studies have shown that the T allele of NPSR rs324981 is associated with increased anxiety sensitivity and an intensified fear response [83–85]. Additionally, individuals carrying the NPSR A allele, especially women, exhibit heightened sensitivity to familial relationships and a greater likelihood of developing mood and anxiety disorders in negative familial contexts. These findings suggest that the effects of NPSR variants are modulated by environmental interactions and display gender-specific characteristics [86–88]. Researchers have employed NPSR knockout mice to explore the biological role of the NPS–NPSR system. Liu et al. [89] utilized NPS−/−/NPSEGFP double transgenic mice, bred with C57BL/6J females, and found that NPSR (−/−) mice exhibited reduced exploration of the central area of the open field and the open arm region, indicating that NPSR deletion heightened anxiety-related behaviors. Duangdao et al. [90] generated NPSR (−/−) mice on a 129S6/SvEvTac background and discovered that NPS (0.1 nmol, i.c.v.) significantly enhanced spontaneous activity in WT mice, while having no effect on NPSR (−/−) mice. Additionally, NPSR (−/−) mice demonstrated a markedly prolonged latency period when transitioning from the dark side to the light box in the dark/light box test. Ruzza et al. [91] used NPSR (+/+)/NPSR (−/−) mice with a CD-1 genetic background and found that NPS (1 nmol, i.c.v.) exhibited anxiolytic and anticonvulsant effects in NPSR (+/+) mice, but not in NPSR (−/−) mice.

#### Role of NPS–NPSR system in pain and implications for PTSD treatment

Pain is typically defined as an unpleasant sensation linked to actual or potential tissue damage, and individuals experiencing pain often contend with negative emotions, such as anxiety and fear, collectively referred to as pain-related emotions [92]. Patients diagnosed with PTSD frequently encounter pain-related challenges, particularly pain-related anxiety. Research indicates that chronic pain and PTSD can mutually exacerbate one another, leading to intensified symptoms of both conditions [93]. In examining the mechanisms of NPS in pain regulation, it has been demonstrated that NPS promotes antinociceptive behavior in rats by activating NPSR and inducing downstream phosphorylation of extracellular signal-regulated kinase 1/2 (ERK1/2) in a subpopulation of pyramidal neurons located in the MeA. Additionally, NPS inhibits hyperpolarization-activated cyclic nucleotide-gated channel currents in the rat amygdala [94]. The MeA is integral to the consolidation of fear memories—a hallmark of PTSD—as well as the affective dimension of pain [95]. Therefore, the NPS-mediated regulation of MeA pyramidal neurons may contribute to the modulation of pain processing and the alleviation of PTSD-related symptoms. Moreover, NPS can influence the output of the amygdala and modify pain-related affective behaviors by enhancing the postsynaptic activity of specific clusters of inhibitory intercalated cells in a protein kinase A (PKA)-dependent manner [96]. In individuals with PTSD, the functionality of the amygdala’s inhibitory intercalated cells is compromised [97]. Thus, the enhancement of these cells’ activity by NPS may not only reduce pain-related anxiety but also normalize amygdala overactivation in PTSD, suggesting a dual role in alleviating both comorbid symptoms.

Furthermore, NPS has been shown to enhance the effects of electroacupuncture in suppressing pain and associated anxiety-like behaviors, potentially linked to increased expression of the NPS/NPSR system in the anterior cingulate cortex [98]. In a formalin assay conducted with mice, Holanda et al. [99] observed that SCH 23390, a selective DA D1 antagonist, slightly diminished the analgesic effects of NPS during the second phase. In contrast, haloperidol, a nonselective DA D2-like receptor antagonist, significantly inhibited the NPS-induced antinociceptive effects in both phases. These findings indicate that the mechanism by which NPS alleviates formalin-induced nociceptive responses involves both D1 and D2 receptors, with a predominant role attributed to D2 receptors. Previous studies have implicated DA D2 receptors in the reward deficiency and hypervigilance observed in PTSD [100, 101]. Consequently, the regulation of D2 receptors by NPS may align pain relief with the alleviation of PTSD-related symptoms. Moreover, NPS (10 nmol, i.c.v.) produces analgesic effects in the hot plate test, mediated by LC noradrenergic activity, as demonstrated by the correlation between NPS-induced hot plate latency and noradrenaline levels in the cerebral cortex, primarily sourced from the LC [102]. Thus, NPS may interact with noradrenergic neuronal activity in the LC to facilitate pain alleviation. Additionally, the adenosine A2A receptor antagonist ZM241385 (0.01 nmol, i.c.v.) blocked the analgesic effects of NPS in the formalin test, while the adenosine A1 receptor antagonist DPCPX (0.001 nmol, i.c.v.) inhibited NPS effects only in the first phase. These results suggest that the central antinociceptive effects evoked by NPS are mediated by the activation of A1A and A2A receptors during the first phase of the formalin test, and primarily through A2A receptors in the second phase [103]. NPS (0.3 nmol and 1 nmol, i.c.v.) also induced antinociception in a restraint stress-induced analgesia mouse model. This effect was reversible through intra-ventrolateral periaqueductal gray (vlPAG) microinjection of antagonists targeting orexin-1 receptors, neurokinin-1 receptors, metabotropic glutamate receptor 5, and cannabinoid receptor 1, suggesting that NPS exerts analgesic effects in stress-induced analgesia via a sequential cascade mediated by orexin-1 receptor-neurokinin-1 receptor-metabotropic glutamate receptor 5-cannabinoid receptor 1 during the stress response in PTSD [104]. While direct evidence remains insufficient to confirm that the NPS–NPSR system modulates chronic pain induced by PTSD, the established role and mechanisms of NPS in pain alleviation indicate that NPSR could represent a promising therapeutic target for addressing both chronic pain and associated anxiety symptoms stemming from PTSD.

#### Role of NPS–NPSR system in substances use disorder and implications for PTSD treatment

There is a significant relationship between PTSD and substance use disorders, as PTSD symptoms have been shown to elevate the likelihood of alcohol and drug use in the general population [105]. Notably, individuals often resort to alcohol consumption as a coping mechanism for PTSD symptoms, influenced by their expectations and motivations related to alcohol use. Furthermore, a positive correlation exists between the severity of PTSD and hazardous drinking behaviors [106]. Research has demonstrated that the NPS and its receptor (NPSR) system modulate various stages of drug addiction, particularly withdrawal and relapse, although the effects on specific addictive substances vary considerably. Badia-Elder et al. [107] found that the infusion of NPS (0.075, 0.3, 1.2 nmol, i.c.v.) prior to testing significantly reduced 2-h ethanol intake in alcohol-preferring rat strains, with no effect observed in non-preferring control strains. Similarly, Cannella et al. [108] reported that the administration of NPS (0.1, 0.5, 1.0, or 2.0 nmol/rat, i.c.v.) five minutes before an alcohol self-administration session resulted in decreased alcohol consumption among alcohol-preferring rats, while showing no effect in non-preferring rats. Notably, the administration of NPS (1.0, 2.0, or 4.0 nmol, i.c.v.) [109] prior to behavioral testing significantly enhanced cue-induced reinstatement of alcohol-seeking behavior, evidenced by increased alcohol recovery, craving, and relapse-like responses. These effects may be mediated by the activation of the hypocretin-1/orexin-A (Hcrt-1/Ox-A) system, as this effect was blocked by the hypocretin-1/orexin-A antagonist SB-334867 [109, 110]. Laas et al. [24] identified the SNP 324981 in NPSR as being associated with AUD and alcohol consumption in a sex-, environment-, and age-dependent manner. Females carrying the A allele exhibited higher rates of AUD and harmful drinking, while males with the T allele showed increased alcohol consumption and incidence of AUD during ages 15–18 years. Opioids, such as morphine, exert potent sedative and analgesic effects but are also associated with strong addictive side effects [29]. In conditioned place preference (CPP) experiments, chronic morphine treatment led to a significantly high CPP score in mice. Li et al. [111] found that administration of NPS (0.3–10 nmol, i.c.v.) alone did not elicit a preference or aversive response in mice; however, NPS treatment combined with morphine reduced both the acquisition and expression of morphine-induced CPP. Additionally, NPS (1.0 nmol, i.c.v.) effectively alleviated anxiety-like behaviors in morphine-abstinent rats. Following acute (12 h) and protracted (7 days) withdrawal from morphine, NPSR expression increased in the VTA and the BLA, while it decreased in the bed nucleus of the stria terminalis (BNST) after seven days of withdrawal [29]. Consequently, researchers postulated that the NPS–NPSR system may play a regulatory role in morphine dependence behaviors.

Cocaine, a stimulant drug, exhibits contrasting effects compared to NPS in morphine dependence. Activation of NPSR by NPS has been shown to increase cocaine-seeking behavior [112] and motor excitability [113]. Chou et al. [114] discovered that NPS enhanced the reinstatement of cocaine-induced CPP in mice, while blocking NPSR mitigated restraint stress-induced cocaine CPP. The study further indicated that the effects of NPS on cocaine seeking and relapse are linked to the activation of orexin neurons in the lateral hypothalamus (LH) and orexin release in the VTA, with orexin receptor 1 (OXR1) and cannabinoid receptor 1 (CBR1)-mediated signaling involved in the function of NPS [114]. Kallupi et al. [112, 115] found that NPS activated Hcrt-1/Ox-A neurons in the LH and perifornical area (PeF), which may project to the VTA and other mesolimbic regions, thereby promoting cocaine-seeking behavior in rats. Administration of NPS in the LH and PeF increased conditioned reinstatement of cocaine seeking, while NPSR antagonists NPSR-QAA1 and [D-Cys(Tbut)5] NPS significantly reduced cue-induced cocaine-seeking behavior.

#### Role of NPS–NPSR system in cognitive impairment and implications for PTSD treatment

PTSD is significantly correlated with cognitive impairments, which include deficits in attention, executive function, and memory. These cognitive deficits are intricately linked to the processes of fear learning and extinction [116]. Numerous studies have shown that individuals with PTSD exhibit an exaggerated response to fear memories, frequently accompanied by impairments in attention and memory. The NPS–NPSR system is increasingly recognized as a modulator of cognitive processes. For example, NPSR or NPS knockout mice exhibit reduced prepulse inhibition (PPI) [117], a phenomenon potentially linked to attention deficits that resemble the attentional hypervigilance observed in PTSD [118]. Additionally, genetic variation in NPSR, specifically the T allele of rs324981, is associated with higher total scores for attention deficit hyperactivity disorder (ADHD) symptoms in humans. Homozygous individuals demonstrate greater sensitivity to environmental factors, such as stress and anxiety, compared to heterozygous individuals [88]. Furthermore, the frontal cortex of spontaneously hypertensive rats (SHR), a model for ADHD, shows significantly reduced NPS levels and increased DA uptake activity compared to control strains [119]. Intraventricular injection of NPS in mice enhanced long-term memory in a dose-dependent manner, as evidenced by improved performance in inhibitory avoidance and novel object recognition tasks [120]. Additionally, NPS administration facilitated spatial learning and memory in the Morris water maze experiment [121]. Similarly, nasal administration of NPS in rats demonstrated enhanced object memory in object discrimination paradigms [122] and promoted cognitive flexibility in the reversal learning test using the T-Maze [123]. Pretreatment with the NPSR antagonist SHA68 has been shown to diminish the memory-enhancing effects of NPS [89], [120]. Wang et al. [124] identified endogenous NPS as a crucial neural regulator of olfactory spatial memory. Administration of [D-Val5] NPS (20 nmol, i.c.v.) alongside SHA68 (10 and 50 mg/kg, i.p.), the NPSR antagonists, resulted in a significant reduction in olfactory spatial memory behavior. This was accompanied by a decrease in the percentage of c-Fos and NPSR immunoreactive neurons within the anterior olfactory nucleus, piriform cortex, subiculum, presubiculum, and parasubiculum [124]. Additionally, NPS has been shown to reverse memory impairments induced by MK801, a selective NMDA receptor antagonist, and scopolamine, a muscarinic cholinergic receptor antagonist [125]. Shao et al. [126] demonstrated that NPS mitigated scopolamine and MK801-induced impairments in olfactory spatial memory, as assessed by a computer-assisted 4-hole-board spatial memory test, by selectively activating NPSR-containing neurons in the subiculum. Research indicates that PTSD is linked to dysregulation of the PFC [127]. Neuropeptide modulation of cortical circuits can influence PFC processing of cognitive and affective behaviors, potentially alleviating cognitive impairments associated with PTSD [128]. Furthermore, the cognitive deficits resulting from sleep restriction were alleviated by NPS (0.1 nmol, i.c.v.), likely due to its capacity to enhance PFC activation and counteract the adverse effects of sleep deprivation [129]. NPS (0.1 nmol, i.c.v.) administered to mice demonstrated a dose-dependent restoration of learning and memory deficits induced by methyl-4-phenyl-1,2,3,6-tetrahydropyridine (MPTP) in the radial arm maze task. This effect may be attributed to the potentiation of glutamatergic synaptic transmission by NPS [130]. Collectively, these findings suggest the potential therapeutic effects of the NPS–NPSR system in the regulation of memory disorders. Although direct evidence linking the NPS–NPSR system to the modulation of PTSD-induced cognitive impairments is currently lacking, the established role of NPS in enhancing attentional processes and facilitating learning and memory indicates that NPSR may serve as a viable target for alleviating cognitive impairment symptoms associated with PTSD.

#### Role of NPS–NPSR system in food intake and implications for PTSD treatment

The prevalence of comorbid PTSD among patients with eating disorders varies between 9% and 24%. Research indicates that the presence of comorbid PTSD is linked to more severe symptoms of eating disorders [131, 132]. Notably, PTSD shows a strong correlation with obesity, impacting approximately 5.8% of U.S. veterans who experience both conditions [133]. This association contributes to considerable psychiatric challenges and an increased risk of suicidal tendencies. Regarding the effect of NPS on food intake, a study conducted by Peng et al. [134] demonstrated that NPS functions as a novel anorectic agent by inhibiting food intake in fasting rats, without altering levels of Neuropeptide Y (NPY) and insulin. Furthermore, additional research indicates that NPS reduces standard food intake in both food-restricted and ad libitum-fed rats. Notably, NPSR antagonists have been shown to counteract the anorexic effects of NPS [135]. Evidence suggests that the PVN in rats is a critical brain region where NPS influences food intake inhibition [136].

## Conclusion

**Figure 1.**
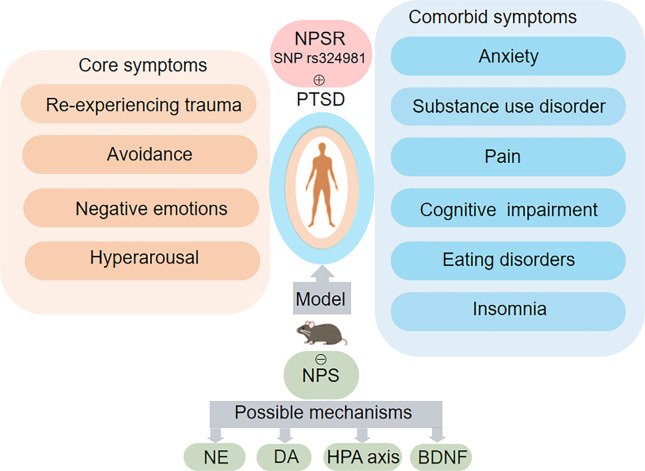
**Role of NPS–NPSR system in PTSD and related neuropsychiatric disorders.** The orange and blue boxes describe the core symptoms of PTSD and related neuropsychiatric disorders, respectively. A variation in the NPSR gene (SNP rs324981) increases the incidence of PTSD in human. Animal models can mimic the core symptoms of PTSD and related neuropsychiatric disorders. Administration of NPS inhibits PTSD through mechanisms involving NE, DA, HPA, and BDNF. Abbreviations: NPS: Neuropeptide S; NPSR: Neuropeptide S receptor; PTSD: Post-traumatic stress disorder; NE: Norepinephrine; DA: Dopamine; HPA: Hypothalamic-pituitary-adrenal axis; BDNF: Brain-derived neurotrophic factor.

**Figure 2.**
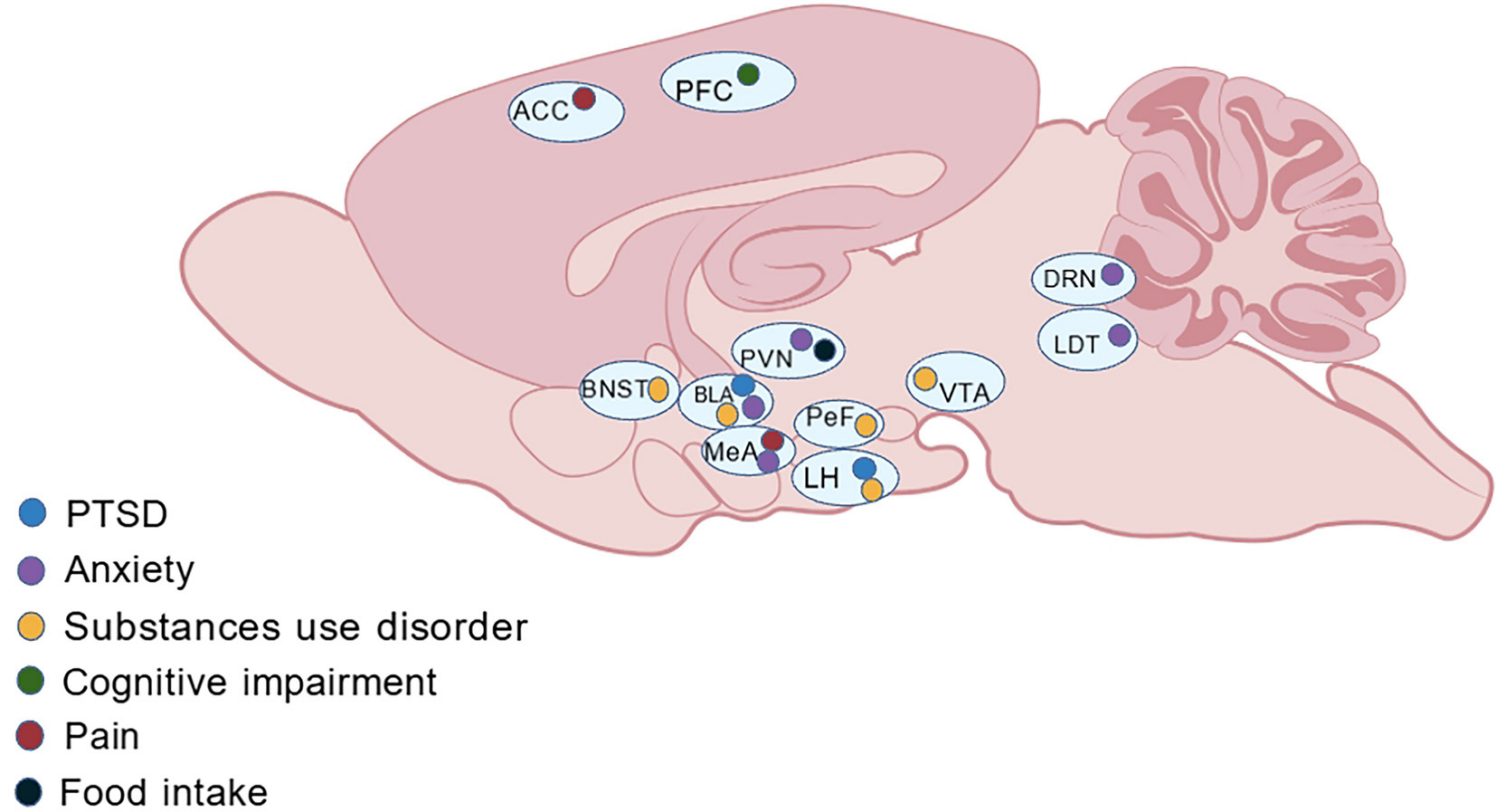
**Brain regions mediate the alleviating effects of NPS on the animal behaviors associated with PTSD and its related neuropsychiatric disorders.** The various colored solid circles displayed in the different brain regions represent the various symptoms that can be relieved by NPS. The Generic Diagramming Platform (GDP), accessible at http://biogdp.com, was employed to create the schematic diagram. Abbreviations: ACC: Anterior cingulate cortex; PFC: Prefrontal cortex; BNST: Bed nucleus of the stria terminalis; BLA: Basolateral amygdala; PVN: Paraventricular nucleus of the hypothalamus; LH: Lateral hypothalamus; PeF: Perifornical area; MeA: Medial amygdala; VTA: Ventral tegmental area; DRN: Dorsal raphe nucleus; LDT: Laterodorsal tegmental nucleus.

NPS–NPSR system plays a potential impact on PTSD and its associated disorder, including anxiety, fear, learning and memory, substances use disorder, eating disorders and pain (Table 1). These effects are mediated by various brain regions (Figure 1) and mechanisms (Figure 2). Based on current preclinical evidence from animal studies, NPSR may serve as a potential target for modulating PTSD responses, offering novel insights into the neural mechanisms underlying PTSD.

However, since the regulatory effects of NPS on neuropsychiatric disorders are exclusively derived from evidence in animal experiments, it remains uncertain whether NPS exerts regulatory effects on PTSD in humans. Therefore, it is necessary to employ animal models with higher validity that can reflect PTSD and its comorbidities to investigate the role of NPS. Concurrently, further research is required to elucidate the mechanisms underlying the actions of NPS and NPSR in PTSD and its comorbidities, such as the molecular and neural circuit mechanisms through which NPSR modulates DA, NE, and the HPA axis in PTSD models.

Limited tools have hindered understanding of the NPS pathway. In recent years, however, techniques such as fluorescent neuromodulator sensors, electrochemical sensors, and *in vivo* microdialysis coupled with mass spectrometry enable real-time monitoring of fluctuations in neuropeptide release presence. Similarly, tools like photoactivatable neuropeptides and genetically encoded biosensors derived from specific nanobodies provided precise spatial and temporal resolution of G protein-coupled receptor (GPCR) activation and inactivation [128], The development of these technologies is accelerating research progress into targeting the NPS–NPSR system for PTSD therapeutic applications.

In addition, regarding NPS, its clinical application is limited by poor metabolic stability and the inability to cross the blood–brain barrier (BBB). Researchers have explored alternative delivery methods, such as nasal administration instead of ventricular injection [137], and developed lipid acylation-modified NPS-palmitic acid self-assembled coupling formulations, which demonstrate effective penetration ability and potential pharmacological effects [138]. Thus, further efforts are warranted to develop optimized delivery systems for NPS. Furthermore, it is imperative to conduct further research targeting NPSR to develop NPSR agonists and antagonists capable of crossing the BBB. In recent years, researchers have rationally designed NPSR agonists exhibiting biased signaling properties. RTI-263, a biased NPSR agonist, demonstrates NPS-like anxiogenic-like effects and memory-enhancing effects in preclinical models. Crucially, and distinct from NPS, RTI-263 significantly attenuates cue-induced reinstatement of cocaine seeking in rats [139]. Thus, the functional benefits conferred by biased NPSR agonists provide a direction for targeting NPSR in the therapeutic development for PTSD.

Additionally, SNP rs324981 play a crucial role in the regulatory effects of the NPS–NPSR system. Therefore, utilizing an NPSR mutant “humanized” animal model is essential to explore the biological and pharmacological effects of NPS in PTSD [140, 141]. This approach could provide valuable insights for developing precise therapeutic strategies for PTSD based on the modulation of the NPS–NPSR system.
